# Emphysematous pyelonephritis, a rare cause of pneumoperitoneum: a case report and review of literature

**DOI:** 10.1186/1757-1626-1-91

**Published:** 2008-08-14

**Authors:** Alexandros Strofilas, Andreas Manouras, Emmanuel E Lagoudianakis, Aikaterini Kotzadimitriou, Apostolos Pappas, Ioannis Chrysikos, Evangelos Menenakos

**Affiliations:** 1 First Department of Propaedeutic Surgery, Hippocrateion Hospital, Athens Medical School, Athens, Greece; 2 Second Department of Surgery, 417 NIMTS-Nosileutiko Idrima Metohikou Tameiou Stratou (Military Veterans' Fund Hospital), Athens, Greece

## Abstract

**Introduction:**

Emphysematous pyelonephritis is a gas-producing necrotizing bacterial infection that involves the renal parenchyma and perirenal tissue.

**Case presentation:**

We report on a case of a 55 year old Caucasian male with no prior medical history presented with left flank pain and malaise. He was diagnosed with emphysematous pyelonephritis, and was successfully treated in our department. The case is presented along with a literature review.

**Conclusion:**

Prompt diagnosis and early treatment is crucial because of the high rate of mortality. Therapeutic modalities and prognostic factors regarding emphysematous pyelonephritis remain controversial.

## Introduction

The first case of gas-forming renal infection was reported in 1898 by Kelly and MacCallum [1]. Since then many names have been used to describe emphysematous pyelonephritis (EPN) such as renal emphysema, pyelonephritis emphysematousa and pneumonephritis [2]. In 1962 Schultz and Klorfein proposed emphysematous pyelonephritis as the preferred designation name, because it stresses the relationship between acute renal infection and gas formation [3].

Emphysematous pyelonephritis is a severe, potentially fatal, necrotizing pyelonephritis with a variable clinical picture ranging from mild abdominal pain to septic shock. The majority of cases occur in diabetics with poor glycemic control while a small percentage may be due to urinary tract obstruction [4,5]. Previous researchers have postulated that vigorous resuscitation and appropriate medical treatment should be followed by immediate nephrectomy [5,6]. However current advances in treatment, allow patients to be treated with percutaneous drainage in combination with broad spectrum antibiotics [4,7,8].

We present a case of emphysematous pyelonephritis in a patient with no prior medical history of diabetes or urinary obstruction that was successfully treated with antibiotics and open drainage.

## Case presentation

A 55 year old Caucasian male with no prior medical history, non-smoker, presented to the emergency department due to left flank pain that was located abruptly, 2 days ago, with progressive aggravation and malaise.

Initial vital signs showed a temperature of 40°C, heart rate of 88 beats per minute, blood pressure of 120/80 mmHg and a respiratory rate of 20 breaths per minute. Physical examination on admission revealed an ill-appearing man, with left-sided costovertebral angle tenderness; he appeared confused and slightly agitated. He had been anuric for 12 hours prior to admission, providing 500 ml of urine after catheterization of his urinary bladder.

Laboratory tests revealed a white blood cell count (WBC) count of 12,100/mm^3 ^with 76% granulocytes, hemoglobin of 15.7 g/dl, platelet count of 173,010/mm^3^, creatinine level of 1.3 mg/dl and urea of 118 mg/dl. Urine analysis demonstrated numerous WBC and gram negative bacilli.

**Figure 1:**
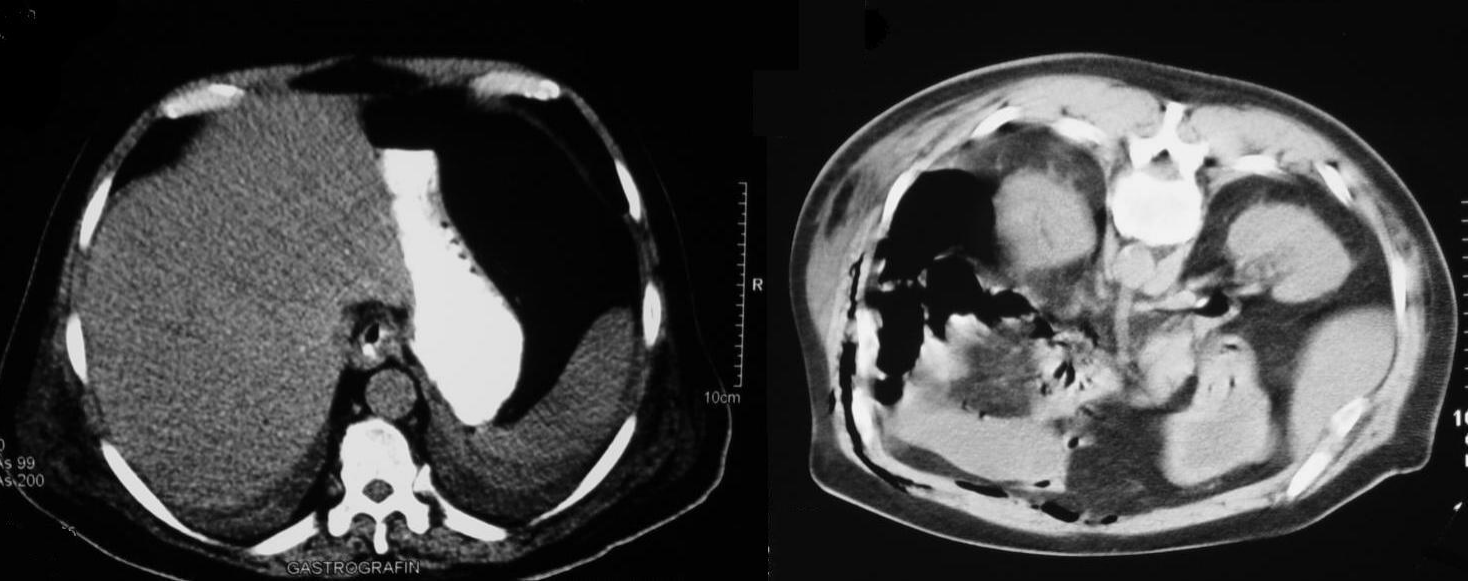
Abdominal CT; intraperitoneal air in the upper abdomen sets the diagnosis of acute abdomen necessitating surgical exploration.

**Figure 2:**
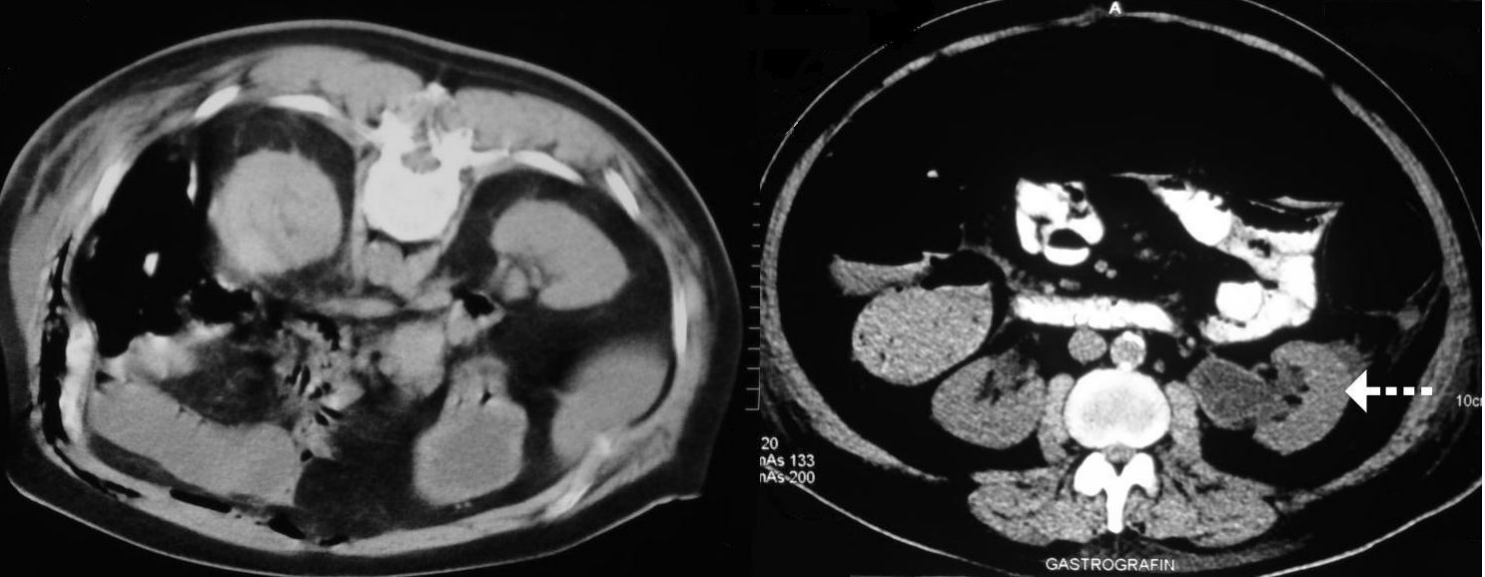
CT scan of the abdomen revealed extensive collections of gaseous fluid mainly in the left retroperitoneum extending along the paracolic gutter and to the middle. The air in the dilated left renal calyces gives the only clue to the diagnosis (arrow).

Ultrasound (US) examination of the abdomen revealed distention of the major calyces and the ureteric pelvis of the left kidney without evidence of urolithiasis. In the following hours the patient slowly deteriorated and became hemodynamically unstable. An abdominal computed tomography (CT) scan took place and the patient was carried to the Intensive Care Unit. Free gas was detected in the intra-peritoneal, as well as, in the extra-peritoneal space (see Figure 1). The extra-peritoneal collected gas was located mostly in the left side of the posterior peritoneal cavity. Moreover, gaseous extension was imaged in the collecting system of the left kidney, without obvious obstruction (see Figure 2).

At laparotomy an extra-peritoneal abscess, located in the left perinephric area, was found and treated with drainage. The patient was treated with intravenous ticarcillin – clavulanic acid (5^a^+0.2^b^) g/vial (TIMENTIN/Smith Kline Beecham, Athens, Greece), 4 times per day, for 12 days. Cultures from the blood and urine sample showed the offensive microorganism to be Escherichia coli. The patient had an uneventful postoperative course and during his hospital stay his symptoms resolved completely.

## Discussion

Emphysematous pyelonephritis has been defined as a necrotizing infection of the renal parenchyma and its surrounding areas that results in the presence of gas in the renal parenchyma, collecting system or perinephric tissue [4]. More than 90% of cases occur in diabetics with poor glycemic control. Other predisposing factors include urinary tract obstruction, polycystic kidneys, end stage renal disease and immunosupression [4,5].

The pathogenesis of EPN remains unclear however four factors have been implicated, including gas-forming bacteria, high tissue glucose level (favoring rapid bacterial growth), impaired tissue perfusion (diabetic nephropathy leads to further compromise regional oxygen delivery in the kidney resulting in tissue ischemia and necrosis; nitrogen released during tissue necrosis) and a defective immune response due to impaired vascular supply. Intrarenal thrombi and renal infarctions have been claimed to be predisposing factors in non-diabetic patients [4,5].

The main bacteria causing emphysematous pyelonephritis are the classical germs of urinary tract infection. The most common is Escherichia coli. Other bacteria include Klebsiella pneumoniae, Proteus mirabilis and Pseudomonas aeruginosa [4-7]. Anaerobic infection is extremely uncommon [9].

The mean patient age is 55 years old. Women outnumbered men probably due to their increased susceptibility to urinary tract infections. The left kidney was more frequently involved than the right one [4].

The clinical manifestations of EPN appear to be similar to those encountered in classical cases of upper urinary tract infections. According to Huang and Tseng [4] fever was encountered in 79% of the patients, abdominal or back pain in 71%, nausea and vomiting in 17%, lethargy and confusion in 19%, dyspnea in 13% and shock in 29%. Laboratory testing revealed elevated glycosylated hemoglobin in 72%, leukocytosis in 67%, thrombocytopenia in 46% and pyuria in 79%. This data comes to agreement with those generally reported in the literature [5-12].

Various imaging techniques can be used to detect gas within the genitourinary system. Ultrasound is insensitive for the diagnosis of renal gas, but useful in diagnosing urinary tract obstruction. It is also a readily available, non-invasive method that is quite useful in the hands of experienced practitioners [11]. Non-contrast CT scan remains the diagnostic method of choice. In addition to showing the presence of gas, it defines the extent of the infection and can diagnose any obstruction [4,5].

Two staging systems, based on CT findings, have been proposed for prognostic and therapeutic reasons. Wan et al [13] described two types. Type I included patients showing parenchymal destruction with streaky or mottled gas but with no fluid collection. These patients had a mortality rate of 69%. Type II patients had renal or perirenal fluid collections that contained bubbly or loculated gas or gas within the collecting system. The mortality rate in this group was 18%. Huang and Tseng et al defined four classes. In class1, gas was limited in the collecting system. In class2, gas was in the renal parenchyma without extension to the extrarenal space. In class 3A, gas extended to the perinephric space, in class 3B, to the pararenal space. Class 4, was referred to bilateral emphysematous pyelonephritis or a solitary kidney with emphysematous pyelonephritis [4,8].

## Conclusion

The treatment of EPN remains controversial. According to some investigators [5,6] vigorous resuscitation, administration of antimicrobial agents and control of blood glucose and electrolytes should be followed by immediate nephrectomy. Huang and Tseng et al [4,8] proposed certain therapeutic modalities based upon their radiological classification system. Localized emphysematous pyelonephritis (class 1 and 2) is confronted by antibiotic treatment, combined with CT-guided percutaneous drainage. For extensive EPN (classes 3 and 4) without signs of organ dysfunction antibiotic therapy combined with percutaneous catheter placement should be attempted. However nephrectomy should be promptly attempted in patients with extensive EPN and signs of organ dysfunction.

Risk factors indicating poor prognosis include thrombocytopenia, acute renal failure, disturbance of consciousness and shock [4,14]. However Falagas et al [15] suggested that increased serum creatinine level, disturbance of consciousness and hypotension may need further research to confirm their potential use as risk factors for fatal outcome. Furthermore their meta-analysis suggest that conservative treatment alone is a risk factor for adverse outcome, although one must take into consideration the different scheme, used by the authors of the studies included, when defining terms such as conservative treatment.

In summary, in high risk groups, such as diabetics, presenting with persistent upper urinary tract infection semiology that does not resolve with proper antibiotic treatment, the presence of a severe renal infection such as EPN should be considered. CT-guided percutaneous drainage or open drainage, along with antibiotic treatment, may be a reasonable alternative to nephrectomy. However surgical intervention should not be delayed in patients with extensive disease or in those who do not substantially improve after appropriate medical treatment and drainage.

## Abbreviations

EPN: emphysematous pyelonephritis; WBC: white blood cell count; US: ultrasound; CT: computed tomography.

## Competing interests

The authors declare that they have no competing interests.

## Authors' contributions

AS contributed to data collection, interpretation of data and literature search of the manuscript. AM contributed to final approval, revision and drafting of the manuscript. EEL contributed to data collection, interpretation of data and literature search of the manuscript. AK contributed to literacy search and drafting of the manuscript. AP contributed to data collection, interpretation of data and literature search of the manuscript. IC contributed to final approval revision and drafting of the manuscript. EM contributed to literacy search and drafting of the manuscript. All authors read and approved the final manuscript.

## Consent

"Written informed consent was obtained from the patient for publication of this case report and accompanying images. A copy of the written consent is available for review by the Editor-in-Chief of this journal."
